# Density of tertiary lymphoid structures and their correlation with prognosis in non-small cell lung cancer

**DOI:** 10.3389/fimmu.2024.1423775

**Published:** 2024-08-13

**Authors:** Shuyue Xin, Shuang Wen, Peipei He, Yulong Zhao, Hui Zhao

**Affiliations:** ^1^ Department of Health Examination Center, The Second Affiliated Hospital of Dalian Medical University, Dalian, China; ^2^ Department of Pathology, The Friendship Hospital of Dalian, Dalian, China

**Keywords:** tertiary lymphoid structures (TLS), non-small cell lung cancer (NSCLC), prognosis, tumor immune microenvironment (TIME), nomogram

## Abstract

**Background:**

Tertiary lymphoid structures (TLS), ordered structure of tumor-infiltrating immune cells in tumor immune microenvironment (TIME), play an important role in the development and anti-tumor immunity of various cancers, including liver, colon, and gastric cancers. Previous studies have demonstrated that the presence of TLS in intra-tumoral (IT), invasive margin (IM), and peri-tumoral (PT) regions of the tumors at various maturity statuses. However, the density of TLS in different regions of non-small cell lung cancer (NSCLC) has not been extensively studied.

**Methods:**

TLS and tumor-infiltrating immune cells were assessed using immunohistochemistry (IHC) staining in 82 NSCLC patients. Tumor samples were divided into three subregions as IT, IM and PT regions, and TLS were identified as early/primary TLS (E-TLS) or secondary/follicular TLS (F-TLS). The distribution of TLS in different maturity statuses, along with their correlation with clinicopathological characteristics and prognostic value, was assessed. Nomograms were used to predict the probability of 1-, 3-, and 5-year overall survival (OS) in patients with NSCLC.

**Results:**

The density of TLS and proportion of F-TLS in the IT region (90.2%, 0.45/mm^2^, and 61.0%, respectively) were significantly higher than those in the IM region (72.0%, 0.18/mm^2^, and 39.0%, respectively) and PT region (67.1%, 0.16/mm^2^, and 40.2%, respectively). A lower density of TLS, especially E-TLS in the IM region, was correlated with better prognosis in NSCLC patients. CD20+ B cells, CD3+ T cells, CD8+ cytotoxic T cells, and CD68+ macrophages were significantly overexpressed in the IM region. CD20+ B cells and CD3+ T cells in the IM region were significantly correlated with the density of E-TLS, while no statistically significant correlation was found with F-TLS. The E-TLS density in the IM region and TNM stage were independent prognostic factors for NSCLC patients. The nomogram showed good prognostic ability.

**Conclusions:**

A higher density of E-TLS in the IM region was associated with a worse prognosis in NSCLC patients, potentially due to the inhibition of TLS maturation caused by the increased density of suppressive immune cells at the tumor invasion front.

## Introduction

Lung cancer is the second most common cancer and the leading cause of cancer-related death (1), and non-small cell lung cancer (NSCLC) accounting for more than 85% of all cases (2). In recent years, with more detailed studies of the tumor immune microenvironment (TIME), immunotherapy has become the most promising treatment method for NSCLC. However, despite the potential for prolonged survival in some patients, only 20-40% of NSCLC patients ultimately benefit from immunotherapy (3), and many more patients suffer from poor outcomes due to immune unresponsiveness or drug resistance. Therefore, it is crucial to further explore the characteristics of TIME and seek better directions for NSCLC immunotherapy.

Tertiary lymphoid structures (TLS) were first identified in inflammation-related tissues, named for their structural resemblance to secondary lymphatic organs (4). In the TIME, TLS are ordered structures of tumor-infiltrating immune cells, mainly consisting of T cell (CD3+) colonies surrounding B cell (CD20+) colonies, with macrophages, dendritic cells, stromal cells, and high endothelial venules at the periphery (5–8). It has been reported that the density and structure of tumor-associated TLS may be related to the clinical outcome of patients with various cancers, such as lung cancer, hepatocellular carcinoma, and melanoma, etc (9–11). TLS can exist in different maturity statuses, which influence prognosis differently. Mature TLS, also known as secondary/follicular TLS, appear to have the same germinal center (GC) as secondary lymphatic organs, with an organized follicular dendritic cell (CD21+) network as its characteristic component. The TLS in their mature state are important sites for initiating or maintaining local and systemic B and T cell responses to tumors (12, 13). High densities of mature TLS-containing GC correlate with favorable clinical prognosis in several cancers, including hepatocellular carcinoma (14), colorectal cancer (15), pancreatic cancer (16), pancreatic neuroendocrine tumor (17), NSCLC (18, 19), and oral cancer (20). In contrast, a higher proportion of immature TLS, also known as primary TLS (without GC), located in tumors, may be associated with poor prognosis (19).

The spatial distribution of TLS within tumors plays different roles in anti-tumor immunity and outcomes. For example, in intrahepatic cholangiocarcinoma, more TLS, especially mature TLS in the tumor core, are associated with improved survival and better responses to immunotherapy. However, more mature TLS in the peri-tumoral regions can lead to worse prognoses (21). Two other studies on liver cancer reached the opposite conclusions. Finkin et al. found that the presence of TLS in the intra-tumor region provided energy for the growth of tumor cells and promoted the progression of liver cancer, leading to worse prognosis in patients (22). Li et al. found that only 30% of TLS were present in the tumor, while higher density of TLS was expressed in peri-tumoral tissues, which was significantly correlated with a good prognosis for patients (23). As such, the density and maturity of TLS vary in different regions of liver cancer and display different prognostic implications. It is important to note that TLS also exist at the invasive margin, the front line of tumor invasion and anti-tumor immunity. In several studies based on tumors of the digestive system, higher densities of immune cells such as CD8+ T cells and Treg immunosuppressor cells were found in the IM regions than in the IT and PT regions, suggesting that invasive margin may have important research value in anti-tumor immunity (24–26). Therefore, the density and maturity of TLS in tumors may differ depending on the pathological type and tissue site. However, it remains unclear whether TLS exhibit spatial heterogeneity in NSCLC and their clinical significance (27).

In this study, we investigated the density and the maturity status of TLS in the intra-tumor, peri-tumor, and invasive margins of NSCLC patients. We also assessed the correlation between TLS density and tumor-infiltrating immune cells and explored the relationship between TLS and prognosis. Our aim is to comprehensively analyze the function of TLS, gain a deeper understanding of their role in the progression of NSCLC, and provide important insights for enhancing immunotherapy as well as identifying a new reference marker for predicting prognosis in NSCLC patients.

## Materials and methods

### Clinicopathologic characteristics of patients

This retrospective study included 95 patients with NSCLC who were diagnosed and underwent resection of their primary lung tumors between January 2011 and November 2018 at Dalian Friendship Hospital. All patients were aged 18 or older, had no prior malignant disease, and did not receive preoperative neoadjuvant chemotherapy and/or radiotherapy. Two patients were excluded because of a history of renal clear cell carcinoma and thyroid carcinoma. Additionally, 11 patients were excluded due to inadequate peritumor content for assessment. Consequently, 82 patients were included in the study cohort and subjected to further analyses (**Table 1**). The study protocol was approved by the Institutional Review Board of the hospital (IRB No. KY-2023 (009)-001). As this is a noninterventional retrospective study, informed consent was waived by the IRB. This study was conducted in accordance with the principles of the Declaration of Helsinki.

**Table 1:** Clinicopathologic characteristics of 82 NSCLC patients.

Clinicopathologic characteristics		Number (%)
Age, years		
	< 65	38(46.3%)
	≥ 65	44(53.7%)
Sex		
	Female	29(35.4%)
	Male	53(64.6%)
Histology		
	Squamous cell carcinoma	23(28.0%)
	Adenocarcinoma	59(72.0%)
TNM		
	I	45(54.9%)
	II	20(24.4%)
	III	17(20.7%)
Tumor size		
	<3cm	54(65.9%)
	≥3cm	28(34.1%)
Tumor location		
	left	30(36.6%)
	right	52(63.4%)
Tumor number		
	Solitary	80(97.6%)
	Multiple	2(2.4%)
Lymph node metastasis		
	Yes	13(15.9%)
	No	69(84.1%)
Differentiation		
	Well/Moderate	26(56.5%)
	Poor	20(43.5%)
Smoking		
	Yes	31(37.8%)
	No	51(62.2%)
Ki67		
	positive	77(93.9%)
	negative	5(6.1%)
P53		
	positive	34(48.6%)
	negative	36(51.4)

Clinical and pathological data, including age, sex, smoking history, and tumor size, were retrieved from electronic hospital records. Tumors were staged according to the 8th edition of the Union for International Cancer Control (UICC) tumor-node-metastases (TNM) classification and pathological staging guidelines. Follow-up information was collected through telephone surveys, with the last follow-up conducted in December 2022. A total of 80 patients were followed-up, with a median follow-up time of 59.1 months. Overall survival (OS) was defined as the time from resection to death, loss to follow-up, or the last follow-up date. Recurrence-free survival (RFS) was defined as the time from surgical resection to the diagnosis of recurrence or the last follow-up.

### Immunohistochemistry

Formalin-fixed, paraffin-embedded (FFPE) tumor tissues were retrieved from pathology archives. A consecutive series of tissue specimens were collected from FFPE and prepared at a thickness of 5 µm. Max Vision staining was performed manually. The tissues were deparaffinized and rehydrated in graded ethanol to water. Antigen retrieval was achieved by pressure cooker treatment for 2 minutes, followed by cooling to room temperature. Endogenous peroxidases were inactivated using 0.3% H_2_O_2_/methanol for 15 minutes at room temperature. Sequential tissue sections were incubated overnight with primary monoclonal antibodies, anti-CD47 (A11382, 1:100, ABclonal) and ready-to-use antibodies from MXB Biotechnologies, including anti-CD8 (MAB-0021), anti-CD3 (MAB-0740), anti-CD20 (kit-0001), anti-CD21 (RMA-0811), and anti-CD68 (kit-0026). Subsequently, the slides were washed three times with PBS for 3 minutes each and then incubated with secondary antibody for 15 minutes. Specific signals were visualized with 3,3′-diaminobenzidine (DAB) for 3-10 minutes (controlled under a microscope), and then washed with PBS for 10 minutes. The sections were counterstained with hematoxylin for 20 seconds, dehydrated and mounted. Necrotic areas were excluded from the analysis.

### Quantification of TLS

**Figure 1:**
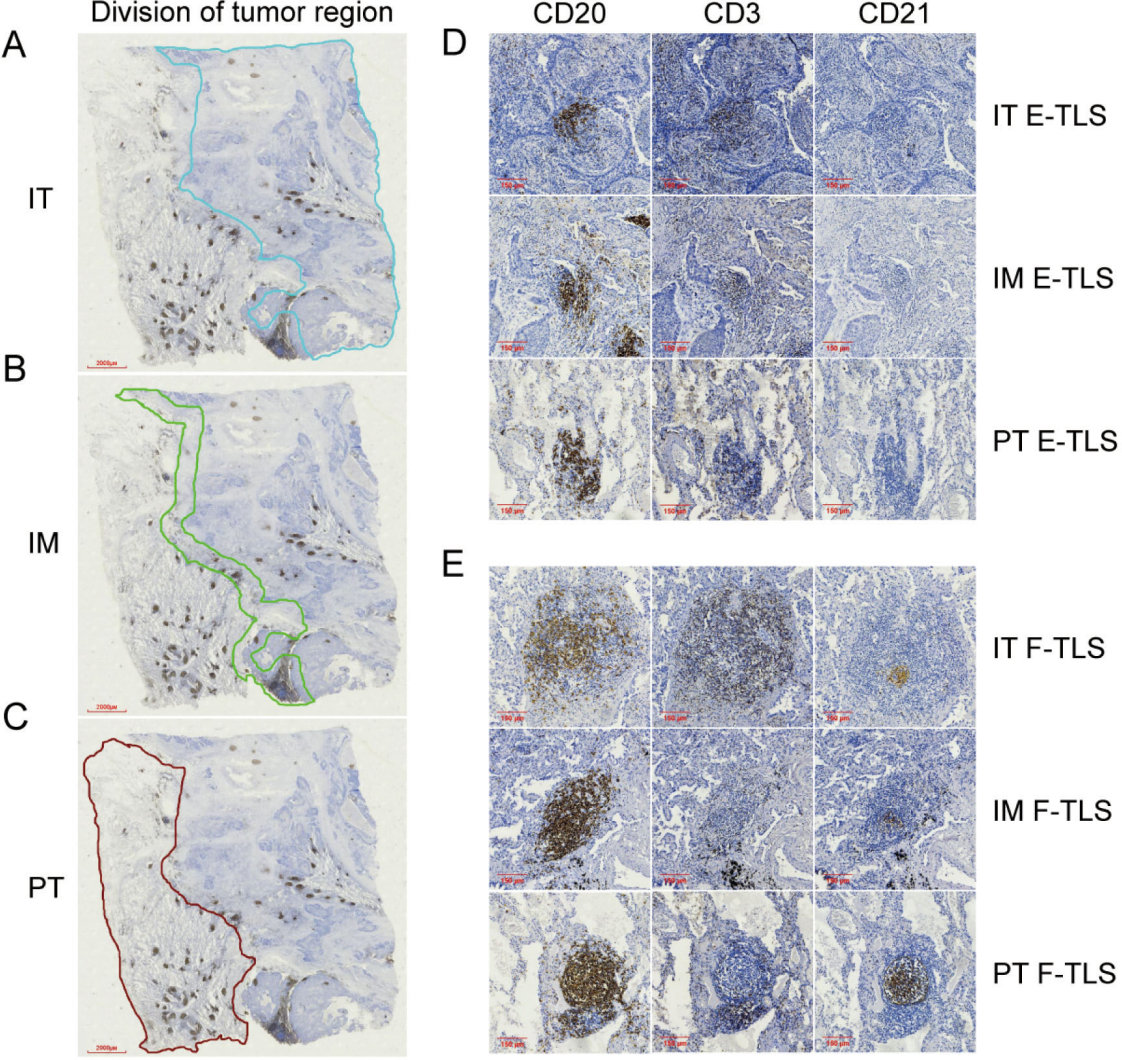
The division of tumor region and the maturity of TLS in subregions. **(A)** The region enclosed by the blue line shown is the IT region (4×). **(B)** The region enclosed by the green line shown is the IM region (4×). **(C)** The region enclosed by the brown lines shown is the PT region (4×). **(D)** Image of E-TLS in IT, IM and PT regions (10×). **(E)** Image of F-TLS in IT, IM and PT regions (10×). IT, intratumor region; IM, invasive margin region; PT, peritumor region; F-TLS, secondary TLS; E-TLS, primary TLS.

TLS were assessed by an expert pathologist (SW) and two observers (SYX and XPH), who were blinded to clinicopathological data. They were trained to identify the pathologic features of TLS in full section slides containing the intra-tumor (IT), invasion margin (IM), and peri-tumor (PT) regions (**Figures 1A–C**). The IM region was defined as 500 μm on each side of the border between the tumor and normal lung tissue (28). TLS was identified by CD3/CD20 staining on consecutive slides for whole FFPE tumor tissue sections (7, 23). A patient was considered TLS-positive (TLS+) if at least one TLS was observed (19). TLS maturity status was categorized into early TLS (E-TLS) and follicle-formed TLS (F-TLS). E-TLS exhibited diffuse lymphocyte aggregation with scarce CD21+ cells (**Figure 1D**), while F-TLS showed follicular morphology with CD21+ follicular dendritic cells (FDCs) (**Figure 1E**) (27, 29). TLS density was calculated as the number of TLS per mm^2^ tissue area in IT, IM, and PT regions (30). ROC curves were constructed based on the overall survival and TLS density, with the maximum Youden’s index used to determine the optimal TLS density cutoff (Youden’s index = sensitivity + specificity -1). Patients were then divided into high and low density groups according to TLS density in each region (23).

### Quantification of tumor-infiltrating immune cells

The density of infiltrating lymphocytes was evaluated by two observers (SYX and XPH) using digital slide review. Digital images were scanned using an MVC3000 slide scanner (Mydream Electronic, Shanghai, China) and quantified using the ImageJ software (NIH). Five randomized microscopic fields (20× magnification) were selected and captured from the IT, IM, and PT regions. Cell density was calculated as the mean number of positive cells per field (23).

### CD47 expression

To further explore immune characteristics, CD47 expression was assessed. Tumor cells express high levels of CD47 during tumorigenesis, binding to macrophage receptors and leading to immune escape (31, 32). The expression rate of CD47 was calculated as the percentage of CD47-positive cells in the corresponding section area (0-100%). Staining intensity of CD47 was categorized as weak (assignment = 1), moderate (assignment = 2), and strong (assignment = 3). The expression score of CD47 was calculated as (32):


$$\begin{array}{l} CD47\;score=\left(\text{percentage of CD}47\text{ stained at weak intensity }\times 1\right)\\ +\;\left(\text{percentage of CD}47\text{ stained at moderate intensity }\times 2\right)\\ +\;\left(\text{percentage of CD}47\text{ stained at strong intensity }\times 3\right) \end{array}$$

Scores ranged from 0 to 300, with a score of 300 indicating 100% strong positive expression in the tumor region.

### Statistical analysis

Statistical analyses were conducted using SPSS 26.0 (IBM) and GraphPad Prism 9 (San Diego, California, USA). Measurement data were expressed as mean ± standard deviation (SD), and counting data were expressed as median (quartile). The t-test or Mann-Whitney U test was used to compare differences in the counting data. Chi-square and continuous correction chi-square tests were used to analyze categorical variables. The optimal TLS density threshold for each subregion was identified using the area under the curve (AUC) of the receiver operating characteristics (ROC) curve. Spearman’s rank correlation was used to analyze the correlations between TLS maturation and immune cells. Kaplan-Meier method was used to plot OS and PFS curves, with the log-rank test for comparison. Prognostic risk for NSCLC patients was assessed using univariate and multivariate Cox regression models. Statistical significance was set as *P*<0.05. Based on multivariate Cox regression analyses, a nomogram including the age, sex, IM E-TLS, and tumor-node-metastasis (TNM) stage was used to predict 1-, 3-, and 5-year overall survival probabilities in NSCLC patients.

## Results

### Density of TLS in different subregions in NSCLC patients

**Figure 2:**
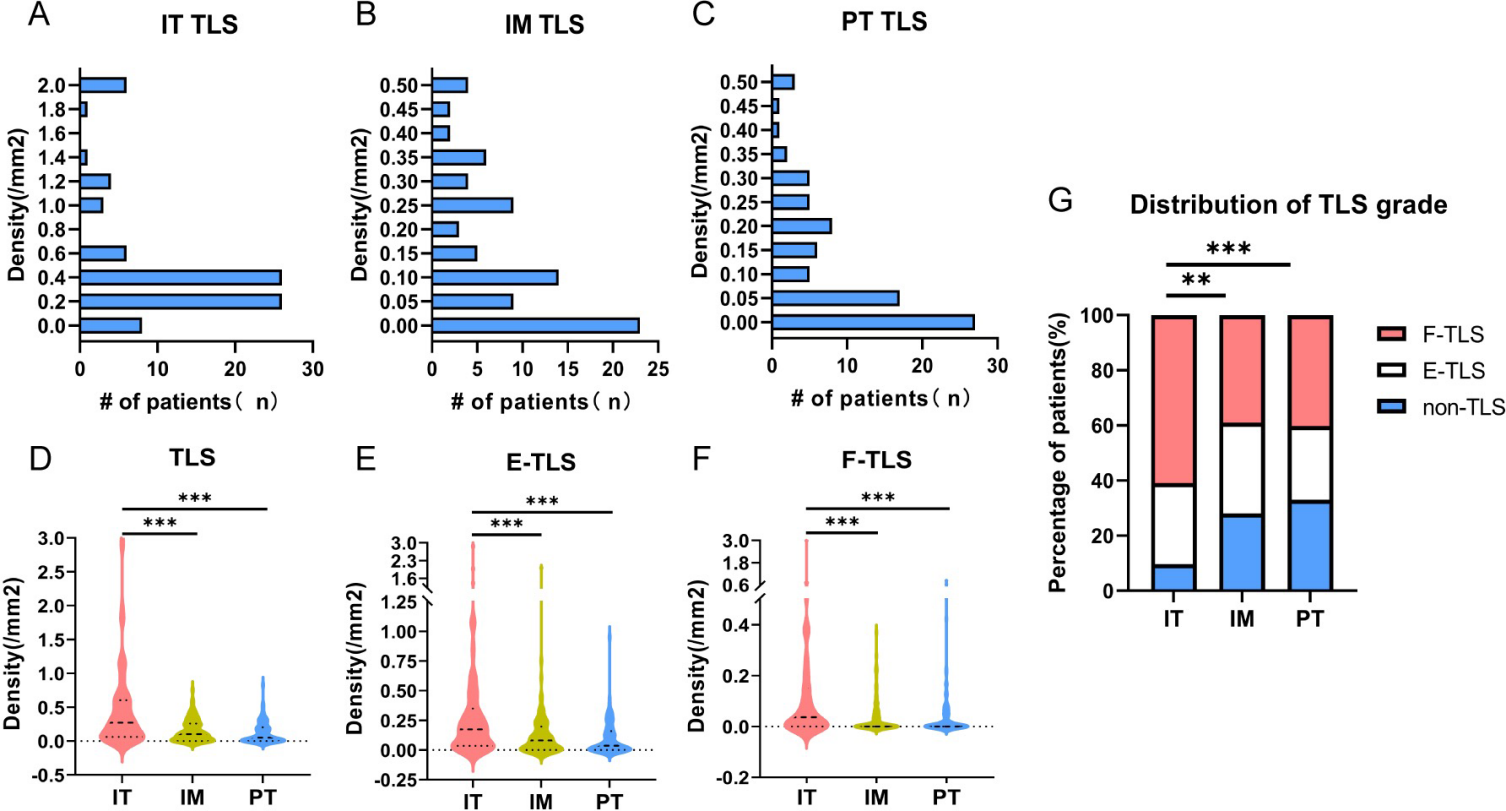
Density of TLS in different maturity grades. **(A)** TLS density and frequency of corresponding patients in IT region. **(B)** TLS density in IM region and frequency of corresponding patients. **(C)** TLS density in PT region and frequency of corresponding patients. **(D)** TLS density and differences in IT, IM and PT regions. **(E)** The density and differences of E-TLS in IT, IM and PT regions. **(F)** The density and differences of F-TLS in IT, IM and PT regions. **(G)** Statistics on the proportion of patients without TLS and with E-TLS and F-TLS in IT, IM and PT regions. IT, intratumor region; IM, invasive margin region; PT, peritumor region; F-TLS, secondary TLS; E-TLS, primary TLS; non-TLS, absence of TLS. ** is *P*<0.01, *** is *P*<0.001.

The presence and location of TLSs were initially assessed in IHC- stained sections of NSCLC patients. Out of the 82 patients, only one had no detectable TLS in the specimen. To further evaluate TLS expression, TLS density was examined in various subregions, revealing significant differences in TLS density frequency. The positive rate of TLS in the IT region (90.2%, 74/82) was significantly higher than in the IM (72.0, 59/82) and PT regions (67.1%, 55/82) (**Figures 2A–C**). As shown in **Figure 2D**, the average TLS density in the IT region (0.45/mm2) was significantly higher than in the IM (0.18/mm2) and PT regions (0.16/mm2) (P<0.00). Regardless of maturity grade, the density was much higher in the IT region (P<0.001, **Figures 2E, F**). Additionally, F-TLS had the highest proportion of TLS in the IT region (61.0%, 50/82) compared to the IM (39.0%, 32/82) and PT regions (40.2%, 33/82; P<0.001, **Figure 2G**).

### Association of TLS and clinicopathologic characteristics of patients

The relationship between TLS density and clinicopathological characteristics was further analyzed (**Table 2**). Higher TLS density in the IT region was detected in tumors with lower TNM stage (*P*=0.002) or no lymph node metastasis (*P*=0.034), suggesting an anti-tumor immune identity for TLS in the IT region. Concurrently, increased TLS density in the IM region was associated with squamous cell carcinoma (*P*=0.005), lymph node metastasis (*P*=0.013), and smoking history (*P*=0.045), while higher PT TLS density was mainly in male patients (*P*=0.001), associated with squamous cell carcinoma (*P*=0.000), large tumor diameter (*P*=0.013), smoking history (*P*=0.004), and absence of P53 (*P*=0.015), which may indicate that TLS density in the IM and PT regions was associated with adverse factors like tumor growth, invasion, and metastasis. These findings suggest that the density of TLS may be involved in various pathways in the different subregions.

**Table 2:** Relationship between clinicopathologic characteristics and density of TLS in NSCLC patients.

Clinical features		Density of IT TLS (/mm2)	*P* value	Density of IM TLS (/mm2)	*P* value	Density of PT TLS (/mm2)	*P* value
Age, years			0.967		0.959		0.842
	<65	1.52		0.21		0.13	
	≥ 65	0.65		0.15		0.18	
Sex			0.684		0.351		0.001
	Male	1.28		0.21		0.19	
	Female	0.63		0.13		0.10	
Histology			0.606		0.005		0.000
	Squamous cell carcinoma	0.73		0.30		0.30	
	Adenocarcinoma	1.18		0.13		0.10	
TNM			0.002		0.125		0.242
	I	1.74		0.18		0.10	
	II	0.26		0.17		0.21	
	III	0.17		0.20		0.25	
Tumor size			0.118		0.051		0.013
	<3cm	1.33		0.14		0.11	
	≥3cm	0.52		0.27		0.23	
Tumor location			0.885		0.546		0.220
	Left	0.56		0.18		0.12	
	Right	1.34		0.18		0.18	
Tumor number			0.489		0.597		0.939
	Single	1.07		0.18		0.16	
	Multiple	0.16		0.09		0.06	
Lymph node metastasis			0.034		0.013		0.323
	Yes	0.16		0.30		0.18	
	No	1.22		0.16		0.15	
Differentiation			0.756		0.238		0.088
	Well/Moderate	0.61		0.19		0.14	
	poor	0.25		0.29		0.24	
Smoking			0.368		0.045		0.004
	Yes	0.78		0.26		0.20	
	No	1.22		0.13		0.13	
Ki67			0.764		0.296		0.450
	Positive	1.09		0.18		0.15	
	Negative	0.46		0.11		0.18	
P53			0.837		0.948		0.015
	Positive	1.6		0.17		0.23	
	Negative	0.81		0.22		0.61	

### Association between density of TLS and prognosis of NSCLC patients

**Figure 3:**
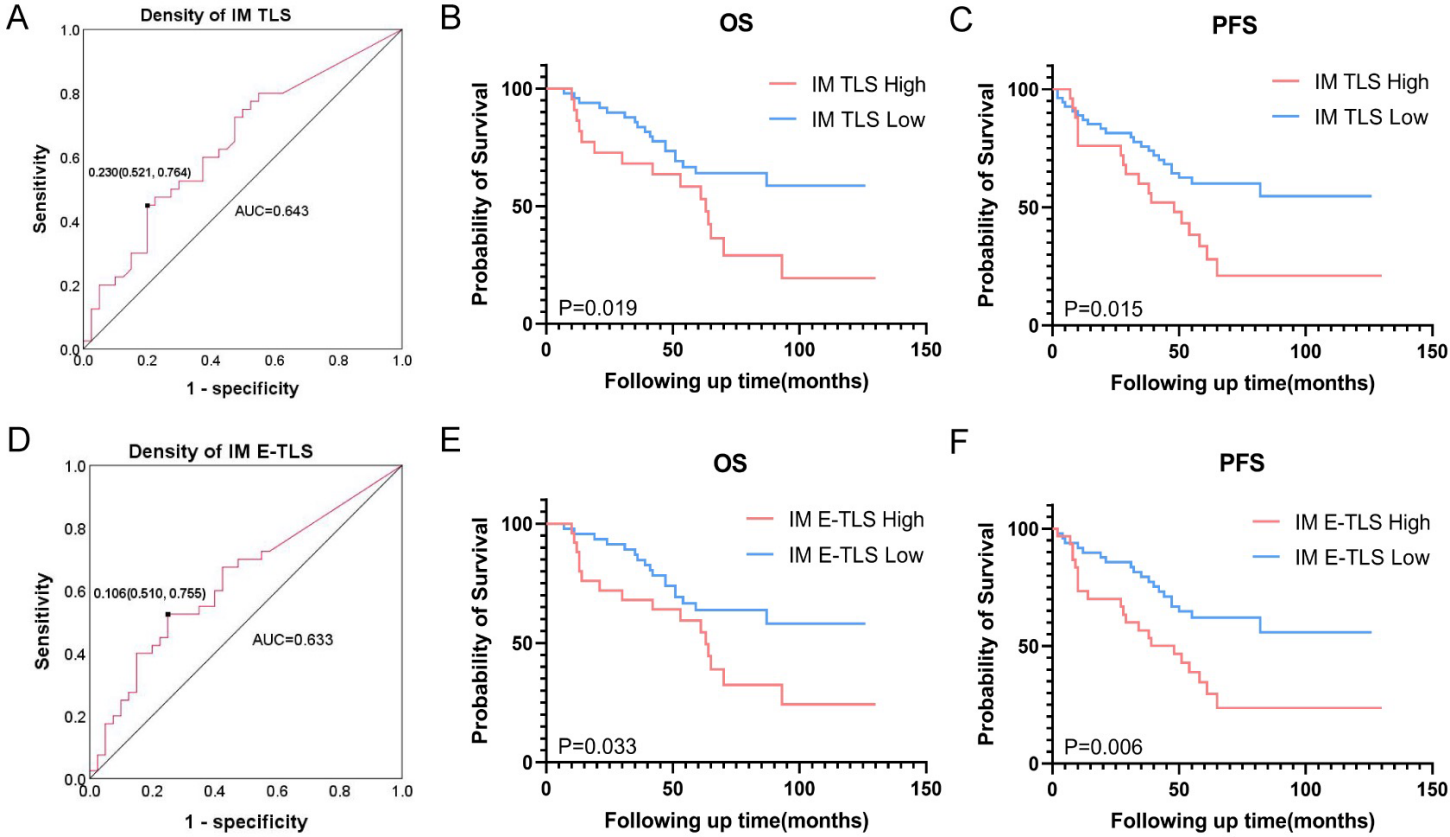
Association between density of TLS and prognosis of NSCLC patients. **(A)** ROC curve based on the overall survival rate of NSCLC patients and TLS density in the IM area, the black dot is the best cut-off value. **(B)** Effect of TLS density in IM region on overall survival. **(C)** Effect of TLS density in IM region on progression-free survival. **(D)** ROC curve based on the overall survival rate of NSCLC patients and E-TLS density in the IM area, the black dot is the best cut-off value. **(E)** Effect of E-TLS density in IM region on overall survival. **(F)** Effect of E-TLS density in IM region on progression-free survival. IT, intratumor region; IM, invasive margin region; PT, peritumor region; F-TLS, secondary TLS; E-TLS, primary TLS.

Given the high positive rates of TLS in the IT, IM, and PT regions (90.2%, 72.0%, and 67.1%, respectively), we first analyzed the impact of TLS presence or absence on patient prognosis but found no clear correlation (**Supplementary Figure 1**). We then conducted a prognostic analysis of the TLS density, stratifying patients into high and low groups based on the optimal cutoff of the ROC. Only the AUC for IM TLS density reached 0.6 (AUC=0.643, 95%*CI*: 0.521-0.764, **Figure 3A**). Kaplan-Meier analysis showed that the low IM TLS group was significantly correlated with longer OS and PFS (**Figures 3B, C**). Considering the different maturity grades of TLS, we further conducted a binary classification of the density of TLS with different maturity grades, revealing that lower density of E-TLS in the IM region was associated with better OS and PFS (**Figures 3D–F**).

### The density of tumor-infiltrating immune cells and its relationship with TLS in subregions

**Figure 4:**
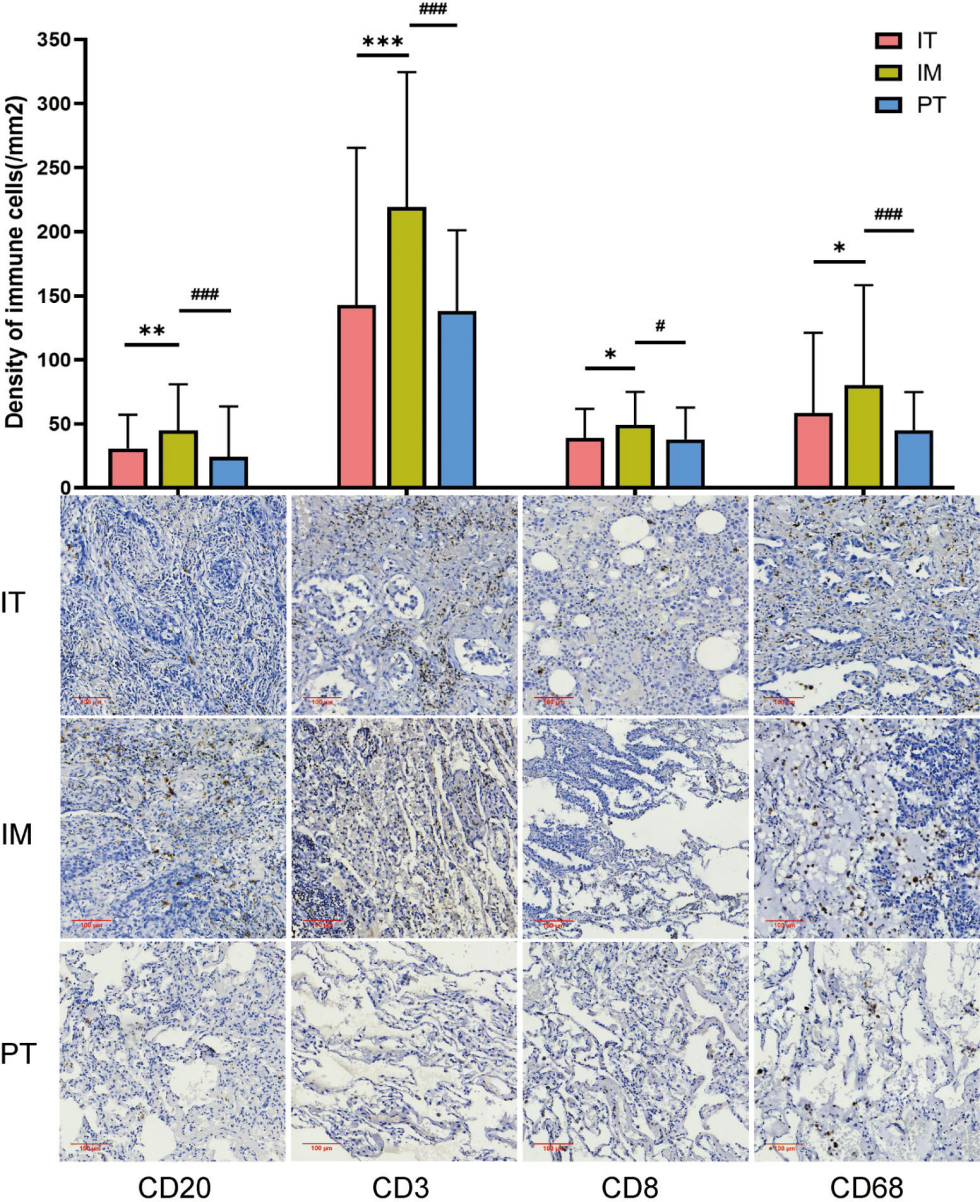
The density of immune cells in different regions. IT, intratumor region; IM, invasive margin region; PT, peritumor region; F-TLS, secondary TLS; E-TLS, primary TLS. *: compared with IT region, *P*<0.05; **: compared with IT region, *P*<0.01; ***: compared with IT region, *P*<0.001; #: compared with IM region, *P*<0.05; ###: compared with IM region, *P*<0.001.

**Figure 5:**
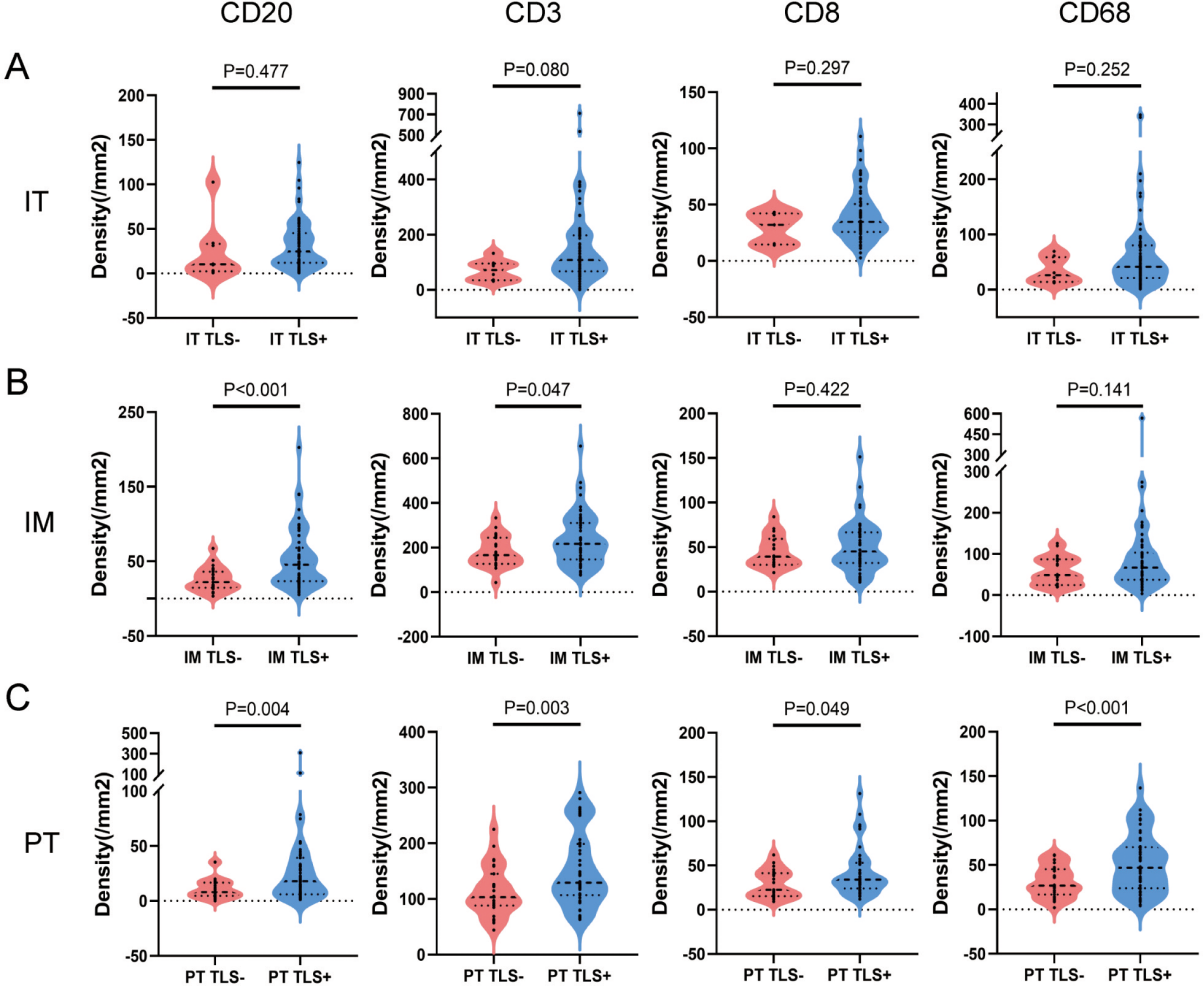
The difference of immune cells between TLS+ and TLS- patients in subregions. **(A)** Patients were divided into IT TLS+ group and IT TLS- group according to the presence or absence of TLS in IT area. Independent sample T test was conducted to analyze the density differences of CD20+ B cells, CD3+ T cells, CD8+ T cells and CD68+ macrophages in IT area between the two groups. **(B)** Patients were divided into IM TLS+ group and IM TLS- group according to the presence or absence of TLS in IM region. Independent sample T test was conducted to analyze the density differences of CD20+ B cells, CD3+ T cells, CD8+ T cells and CD68+ macrophages in IM region between the two groups. **(C)** Patients were divided into the TLS+ group and the TLS- group according to the presence or absence of TLS in the PT area. The density differences of CD20+ B cells, CD3+ T cells, CD8+ T cells and CD68+ macrophages in the PT area were analyzed by independent sample T test. IT, intratumor region; IM, invasive margin region; PT, peritumor region.

TLS structures and tumor-infiltrating immune cells are important components of the TIME, playing crucial roles in tumor immunity. We analyzed the density of immune cells in the subregions of NSCLC tumors, revealing that the density of CD20+ B cells, CD3+ T cells, CD8+ cytotoxic T cells, and CD68+ macrophages was higher in the IM region than in the IT and PT regions (**Figure 4**). Patients were grouped into TLS+ and TLS- categories based on TLS presence in subregions, and differences in immune cells were compared. No significant differences were found between the IT TLS+ and IT TLS- groups (**Figure 5A**). However, the IM TLS+ group had significantly higher densities of CD20+ B cells and CD3+ T cells than the IM TLS- group (*P*=0.000 and *P*=0.047, **Figure 5B**). The PT TLS+ group had significantly higher densities of CD20+ B cells, CD3+ T cells, CD8+ cytotoxic T cells, and CD68+ macrophages than the PT TLS- group (*P*=0.004, *P*=0.003, *P*=0.049, *P*=0.000, **Figure 5C**).

### Association between tumor-infiltrating immune cells and different maturity status of TLS in subregions

**Figure 6:**
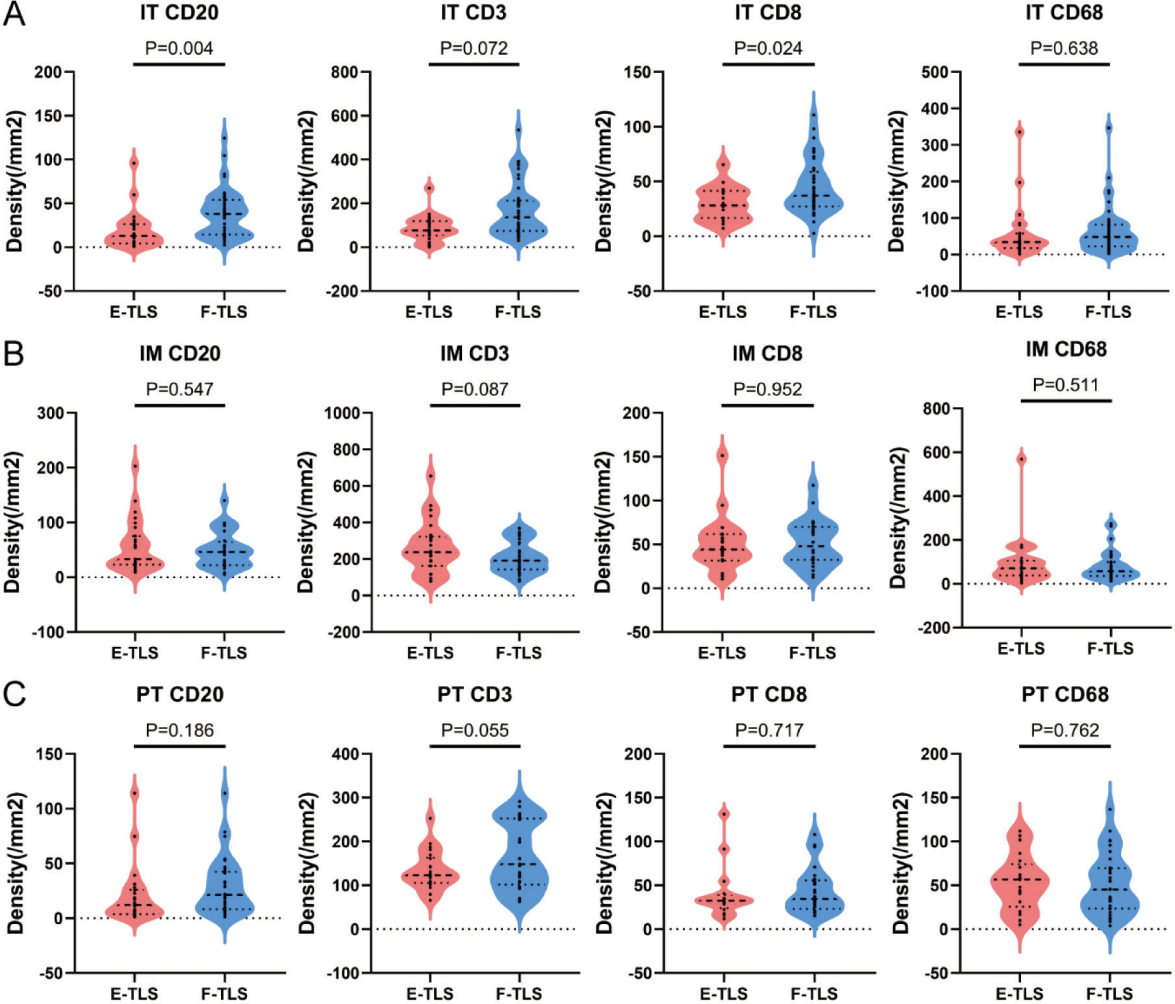
The difference between immune cells with different maturity of TLS. **(A)** Patients were divided into E-TLS group and F-TLS group according to TLS maturation status in IT region. Independent sample T test was conducted to analyze the density differences of CD20+ B cells, CD3+ T cells, CD8+ T cells and CD68+ macrophages in IT area between the two groups. **(B)** Patients were divided into E-TLS group and F-TLS group according to TLS maturation status in IM region. Independent sample T test was conducted to analyze the density differences of CD20+ B cells, CD3+ T cells, CD8+ T cells and CD68+ macrophages in IM region between the two groups. **(C)** Patients were divided into E-TLS group and F-TLS group according to TLS maturation status in the PT region. Independent sample T test was conducted to analyze the density differences of CD20+ B cells, CD3+ T cells, CD8+ T cells and CD68+ macrophages in the PT region between the two groups. IT, intratumor region; IM, invasive margin region; PT, peritumor region; F-TLS, secondary TLS; E-TLS, primary TLS.

We further analyzed the role of tumor-infiltrating immune cells in TLS maturation by comparing immune cell densities between E-TLS and F-TLS groups in subregions. As shown in **Figure 6A**, in the IT region, the densities of CD20+ B cells and CD8+ T cells were significantly higher in the F-TLS group than in the E-TLS group (*P*=0.004 and *P*=0.024, respectively). No statistical difference was observed in the IM and PT regions (**Figures 6B, C**).

### Correlation between TLS and immune cells

**Figure 7:**
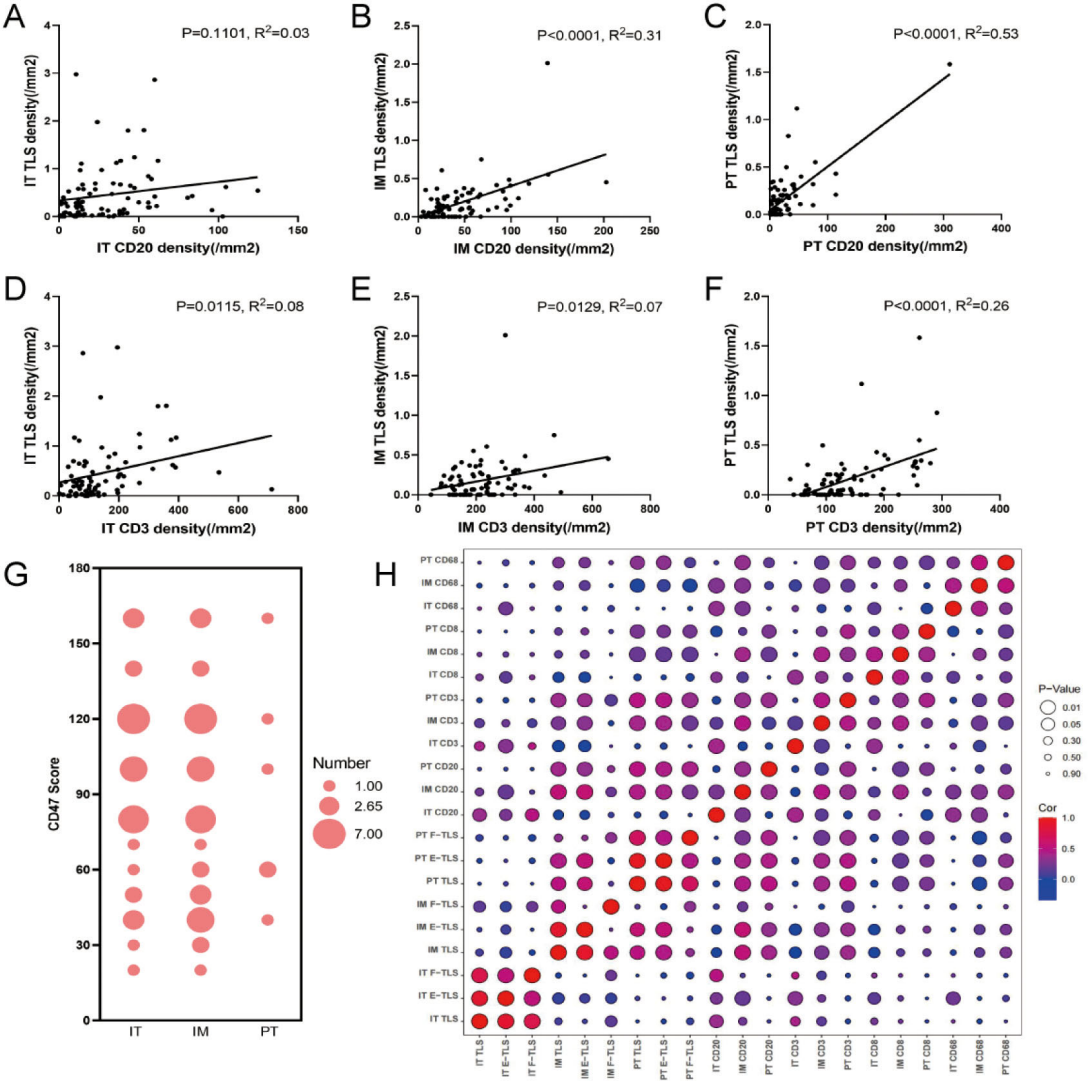
Correlation between the density of immune cells and TLS. **(A–C)** General linear regression analysis was performed to determine the correlation between CD20+ B cells in IT, IM and PT regions and TLS density in the corresponding regions. **(D–F)** General linear regression analysis was performed to determine the correlation between CD3+ T cells in IT, IM and PT regions and TLS density in the corresponding regions. **(G)** The bubble chart shows the CD47 scores in the IT, IM and PT regions (the size of the circle indicates the score frequency). **(H)** The bubble map shows the correlation between TLS at different maturation states in IT, IM and PT regions and immune cells in each region (red indicates positive correlation coefficient, blue indicates negative correlation coefficient, and purple indicates close to 0 correlation coefficient; The larger the circle, the more significant the correlation, and the smaller the circle, the weaker the correlation). IT, intratumor region; IM, invasive margin region; PT, peritumor region; F-TLS, secondary TLS; E-TLS, primary TLS.

We further analyzed the correlation between immune cell densities and TLS density to determine their connection. CD20+ B cell density was significantly correlated with TLS density in the IM and PT regions (*R^2^* = 0.31, *P*<0.0001; *R^2^* = 0.53, *P*<0.0001; **Figures 7A–C**). The correlation was more significant between the E-TLS (**Supplementary Figure 2**). CD3+ T cell density was significantly correlated with TLS density in all three regions (*R^2^* = 0.08, *P*=0.0115; *R^2^* = 0.07, *P*=0.0129; *R^2^* = 0.26, *P*<0.0001; **Figures 7D–F**). Further analysis revealed significant correlations between CD20+ B cells, CD3+ T cells, and TLS density in the three regions, while CD8+ T cells correlated significantly with total TLS and F-TLS density in the IT region, and CD68+ macrophages with total TLS and E-TLS density in the PT region (**Figure 7H**). Prognostic analysis showed that none of the immune cells was associated with prognosis in the IT region. In the IM region, lower density of CD20+ B cells, lower CD8/CD3 ratio, and higher density of CD3+ T cells were associated with better prognosis. In the PT region, lower density of CD20+ B cells was associated with better prognosis (**Supplementary Figure 3**).

CD47 scores, associated with the immune escape, were significantly higher in the IT (mean score 36.34) and IM regions (mean score 39.02) than in the PT region (mean score 6.59), with no significant difference between IT and IM regions (**Figure 7G**). No significant correlation was observed between CD47 scores and TLS density in the IT, IM, and PT regions (**Figure 7H**).

### Density of IM E-TLS as an independent prognostic factor for overall survival

**Figure 8:**
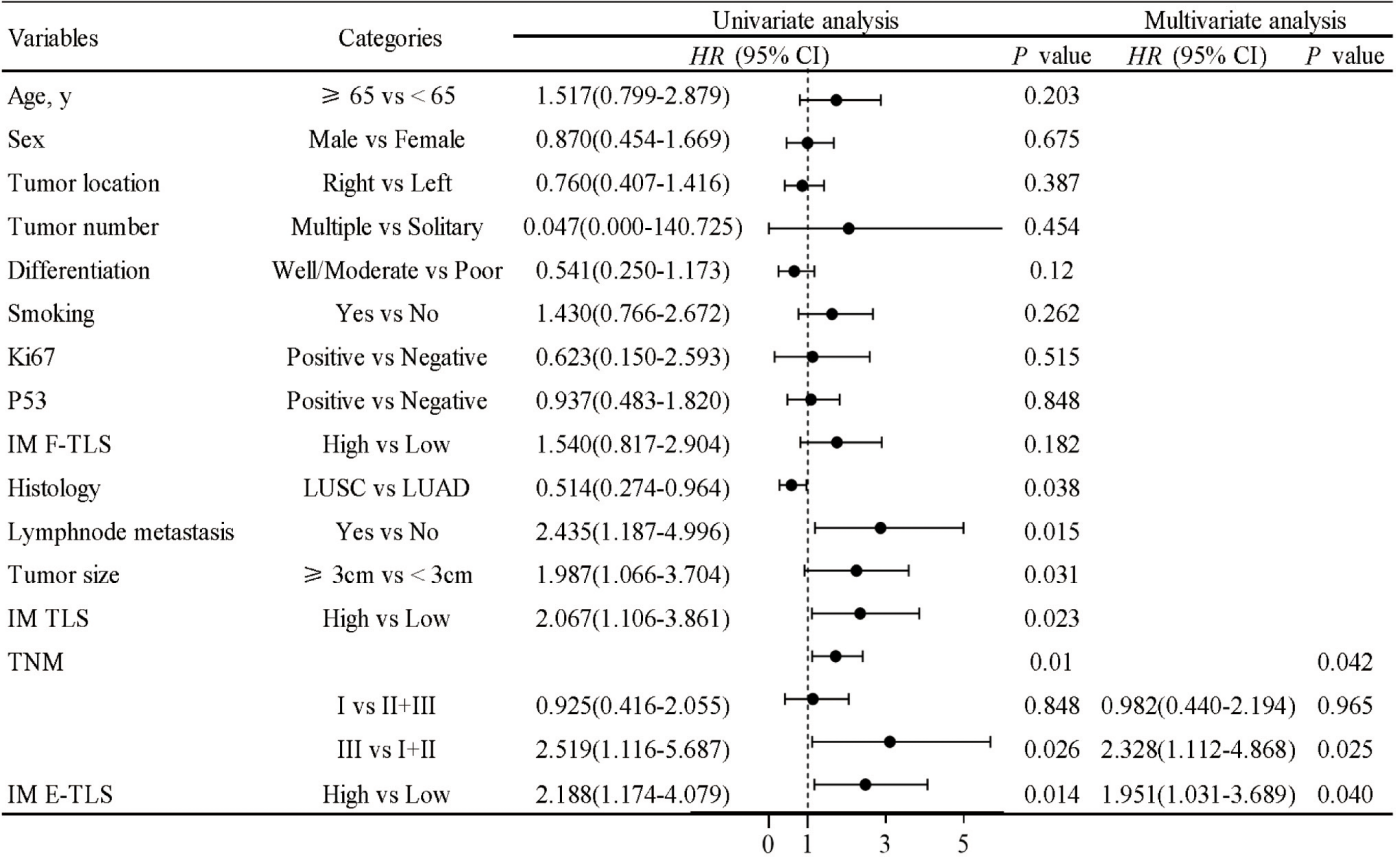
COX proportional hazard regression model for overall survival of NSCLC. A univariate Cox regression analysis was performed on the clinical characteristics and the density of TLS in NSCLC patients. P<0.05 was taken as the threshold to screen out prognostic factors. The relevant factors were included in the multivariate analysis for predicting overall survival (all *P*<0.05).

Univariate Cox regression analysis identified histology, lymph node metastasis, tumor size, TNM stage, and IM TLS and IM E-TLS density as significant prognostic factors. Multivariate analysis confirmed that TNM stage and IM E-TLS can serve as independent prognostic factors for overall survival (all *P*<0.05), while other clinicopathological variables showed no consistent significance (**Figure 8**).

### Nomogram predicted 1-, 3- and 5-year OS in NSCLC patients

**Figure 9:**
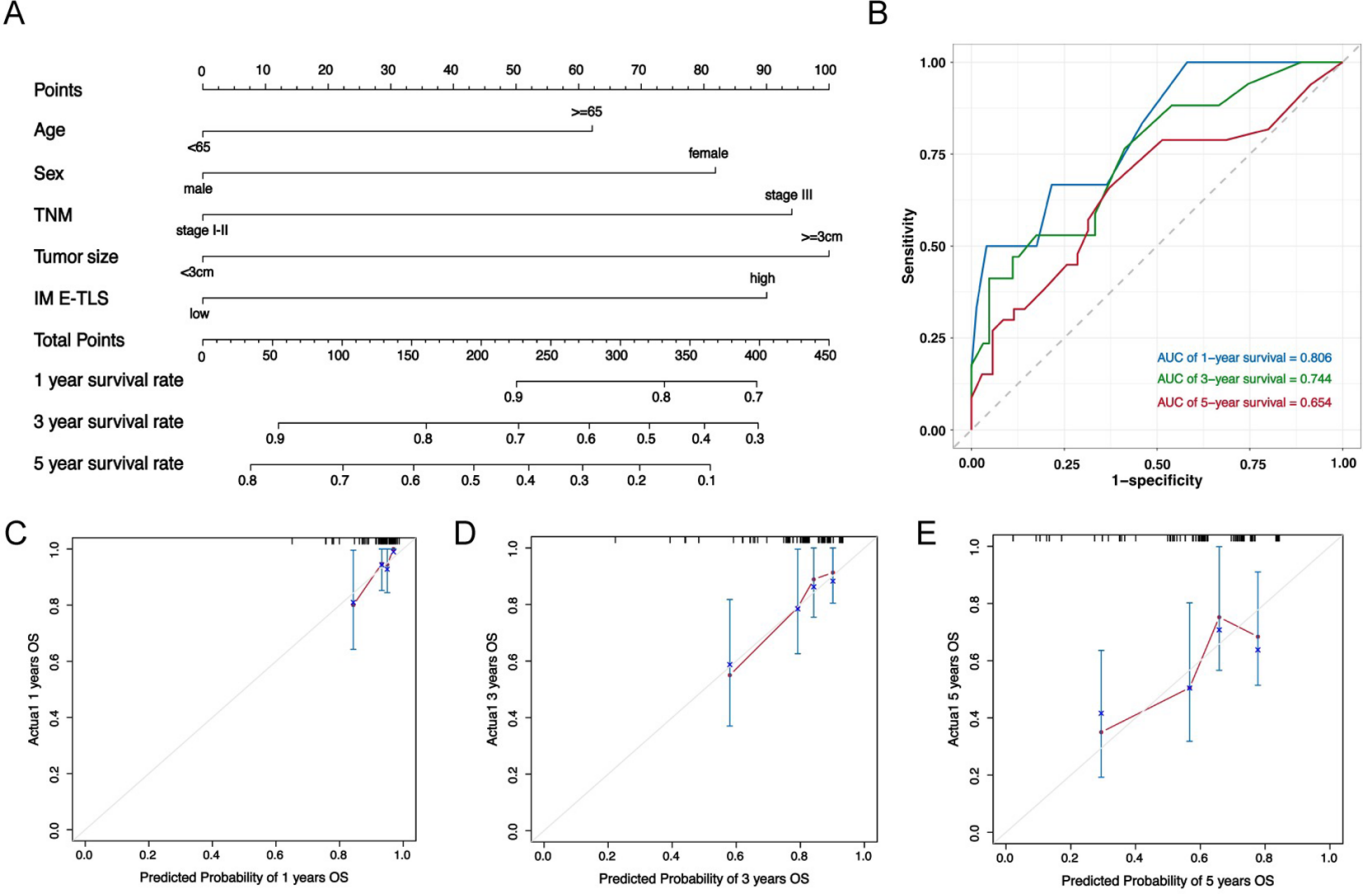
Nomogram predicted the overall survival of NSCLC patients. **(A)** Nomogram was plotted based on four factors: age, sex, density of IM E-TLS and TNM grade, and 1-, 3- and 5-years of OS could be predicted. The probabilities were estimated as the sum of points for each variable as a function of total points. Each variable obtained an integral by drawing a line up from the corresponding value to the ‘point’ line. On the “total points” line, the total sum of points added by each variable was shown. A line was drawn downward to read the associated probability forecasts. The Bootstrap method was used for internal validation, with 200 repeat samples. **(B)** ROC curve to verify nomogram model performance. **(C–E)** Calibration curves of a nomogram to predict OS at 1-, 3- and 5 years in this data set.

Based on multivariate Cox regression analysis, a nomogram prognostic model was established, including IM E-TLS and TNM stage as predictors, along with patient age and sex based on our clinical experience (**Figure 9A**). The nomogram demonstrated good predictive performance for overall survival in NSCLC patients (**Figures 9B–E**).

## Discussion

Our study comprehensively analyzed the density of TLS at different maturity statuses across various subregions and their correlation with the prognosis of NSCLC patients. Moreover, we plotted a nomogram incorporating IM E-TLS, TNM stage, age, and sex to predict patient prognosis. We found a high detection rate of TLS in NSCLC patients (98.8%), with higher density in the IT region compared to the IM and PT regions. Mature TLS had a significantly higher proportion in the IT region than in the other two regions. Based on previous studies, the presence of GC in mature TLS is an important starting point for anti-tumor immune response, we hypothesized that the anti-tumor immunity in the IT region of tumors is stronger than in the other two regions (33).

Our analysis of clinical data from NSCLC patients demonstrated that higher TLS density in the IT region was associated with lower TNM stage and absence of lymph node metastasis, indicating an accumulation of TLS in early-stage NSCLC and better prognosis. By contrast, increased TLS density in the IM and PT regions was associated with adverse prognostic factors such as tumor growth, invasion, and metastasis, suggesting a negative impact on prognosis. Intriguingly, while many studies have argued for a positive prognostic effect of TLS in lung cancer (34, 35), our study found that IT and PT TLS density at any maturity status was not predictive of prognosis. Higher density of TLS in the IM region was associated with worse prognosis in NSCLC patients. Furthermore, high density of E-TLS in the IM region was an independent risk factor for overall survival, possibly due to the absence of a germinal center, rendering TLS less effective (19, 36).

Tumor-infiltrating immune cells may play an important role in TIME and TLS development. We observed the highest proportion of these cells in the IM region, consistent with the findings by Zhu et al. (37). The IM region, as the interface between intra-tumor and peri-tumor tissues, is the front line of tumor invasion. When immune aggregation occurs here, it reflects an active anti-tumor immune response. Although previous studies proposed that the infiltration of immune cells within tumors was higher than that in the peri-tumor region and could be further correlated with better prognosis (38–40), we found higher densities of CD20+ B cells, CD3+ T cells, CD8+ cytotoxic T cells, and CD68+ macrophage cells in the IM region compared to the IT and PT regions, with CD20+ B cell density associated with better prognosis and CD8+/CD3+ ratio with poorer prognosis. We hypothesize that previous studies merging the invasive edge (IM region) with the IT region might have led to different findings. Therefore, in this study, when the IM region was separated from the IT region, a high number of immune cells could be detected in this region.

Given that the IM region had significant association with patient prognosis and obvious immune cell infiltration, our study focused on this region. Just like some of the previous studies, we also found that the density of CD20+ B cells and CD3+ T cells was most closely related to prognosis and immunotherapy response (41–43). These cells were significantly correlated with the presence of TLS, suggesting their role in TLS formation. Notably, the density of CD20+ B cells and CD3+ T cells in the IM region was only positively correlated with the density of E-TLS but not with the presence or density of F-TLS, suggesting that these immune cells are not involved in the maturation of TLS in this region. However, in the PT region, the density of these cells was higher in the tissues in the presence of F-TLS and positively correlated with the density of F-TLS, implying their involvement in TLS development and maturation in peri-tumoral tissues. This contrast highlights that CD20+ B cells and CD3+ T cells participate in TLS formation but are inhibited in TLS maturation in the IM region, leading to high E-TLS density in the IM region.

This study suggests a potential immunosuppressive state in the IM region, based on two main observations. Firstly, the IM region displayed high density of E-TLS. Previous studies have shown that the presence of E-TLS is associated with the immunosuppressive state of the tumor immune microenvironment. When E-TLS is formed in large quantities, immature immune cells often differentiate into immunosuppressive cells (such as regulatory B cells), and in the early stage of disease or after immunotherapy, the production of E-TLS in tumor microenvironment is associated with the expression of immunosuppressive genes and poor prognosis (44–46). CD8+ T cells are the main anti-tumor immune cells associated with favorable prognosis in various tumors, and their immune function can be inhibited by regulatory B cells, which further contributes to the functional suppression of anti-tumor immune cells in the IM region (47, 48). Secondly, we observed a high H-score for CD47 in the IM region. As a marker of immunologically privileged cells, CD47 can be expressed by stromal cells, red blood cells, endothelial cells and other body cells. During tumor progression, high density of tumor cells express CD47 and bind to the corresponding receptors on macrophages to evade the immune system (49). The high density of CD47 in the IM region suggests an immunosuppressive state in this region (50).

Our study demonstrated a correlation between the density of E-TLS in the IM region and prognosis in NSCLC patients, as well as the immune characteristics of the IM region. Studies have found that the IM region of liver cancer is the lesion front for tumor invasion of normal tissues, where the density of immunosuppressive genes is significantly increased (46). Our study found a high density of immune cells and high expression of CD47 in the IM region, suggesting that the anti-tumor immune ability of immune cells may be inhibited, and also proved that the immunosuppressive state of the IM region of NSCLC tumors, and proposed that this immunosuppressive state may be related to the abnormal participation of B cells and T cells in the high density of E-TLS during TLS maturation in the IM region. Currently, it is not clear why large amounts of E-TLS are produced in such early tissue lesions. However, the causal relationship between E-TLS and local immunosuppression remains uncertain. It is also unclear whether E-TLS is the cause or result of local suppression of immune cells in the tissue, whether the mass aggregation of tumor-infiltrating immune cells in the IM region is a manifestation of anti-tumor immunoactivation or compensatory proliferation after immunosuppression, and whether its density can represent the expression and function of cells. These questions require further investigation in future research.

Due to the non-specific early clinical manifestations of NSCLC and the low diagnostic rate, obtaining early tissue samples from patients for research is challenging. We believe that in the future, we can verify our conclusions by multicenter studies and expanding the sample size to discuss the expression and distribution characteristics of TLS in early and advanced NSCLC patients, especially patients at TNM stage I, to clarify the density and distribution characteristics of TLS in the tumor tissues of early NSCLC lesions. Another limitation of this study was the lack of external validation for the nomogram prediction model and the absence of sample data for tumor tripartite in the online database. This again calls for multicenter clinical studies that should be performed to validate our findings. Our study also suggested that future studies should sequence the expression of immune-related genes in the IT, IM, and PT regions. Comparing the upregulation or inhibition of immune-related genes among the three regions, rather than relying solely on immune cell density as a functional indicator, would enable a more comprehensive evaluation of the immunomodulatory effects of tumor-related tissues during tumor development and invasion. This would offer a valuable reference for improving NSCLC immunotherapy.

## Conclusion

In summary, we found that TLS had a high positivity rate in patients with NSCLC. Although TLS had the highest density in the IT region, its density in the IM region was most closely associated with the patient prognosis. A nomogram, including age, sex, IM E-TLS, and tumor-node-metastasis (TNM) stage, was created to predict 1-, 3-, and 5-year overall survival probabilities in NSCLC patients. A higher density of E-TLS in the IM region was associated with poorer prognosis of NSCLC patients, which we believe is linked to the immunosuppressive state in the IM region.

## Data Availability

The raw data supporting the conclusions of this article will be made available by the authors, without undue reservation.
