# Supplementary material for: Density of tertiary lymphoid structures and their correlation with prognosis in non-small cell lung cancer
Source: Front Immunol. 2024 Aug 13;15:1423775. doi: 10.3389/fimmu.2024.1423775 (PMC11347756; doi:10.3389/fimmu.2024.1423775)
Supplement: Supplementary file 1 [file Presentation1.pdf]

Supplementary Material

Expression of tertiary lymphoid structure and its correlation with prognosis in non-small cell lung cancer

Shuyue Xin[1*], Shuang Wen[2*], Peipei He[1*], Yulong Zhao[1], Hui Zhao[1#*]

Correspondence: Hui Zhao: zhaohui@dmu.edu.cn

Supplementary Figures

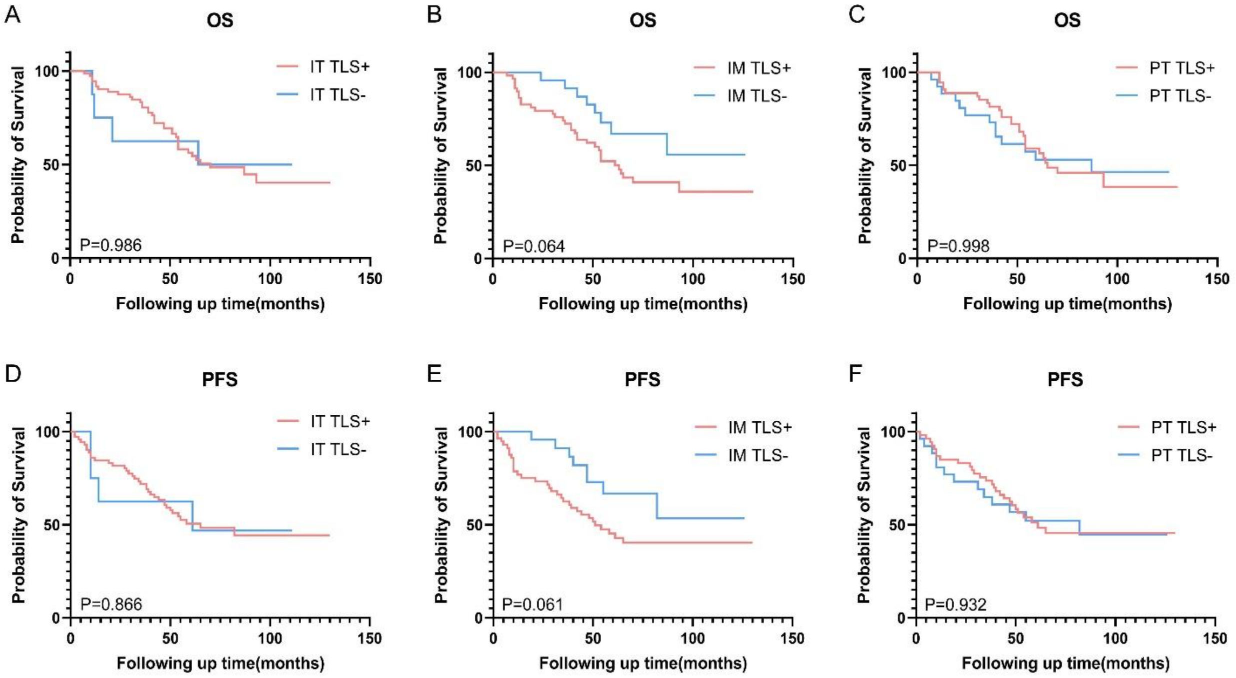

Figure S1. The expression of TLS in subregions and prognosis of NSCLC patients. (A-C) Influence of TLS in IT, IM and PT regions on overall survival by Kaplan-Meier analysis. (D-F) Influence of TLS in IT, IM and PT regions on progression-free survival by Kaplan-Meier analysis. IT, intratumor region; IM, invasive margin region; PT, peritumor region; TLS+, presence of TLS; TLS-, absence of TLS.

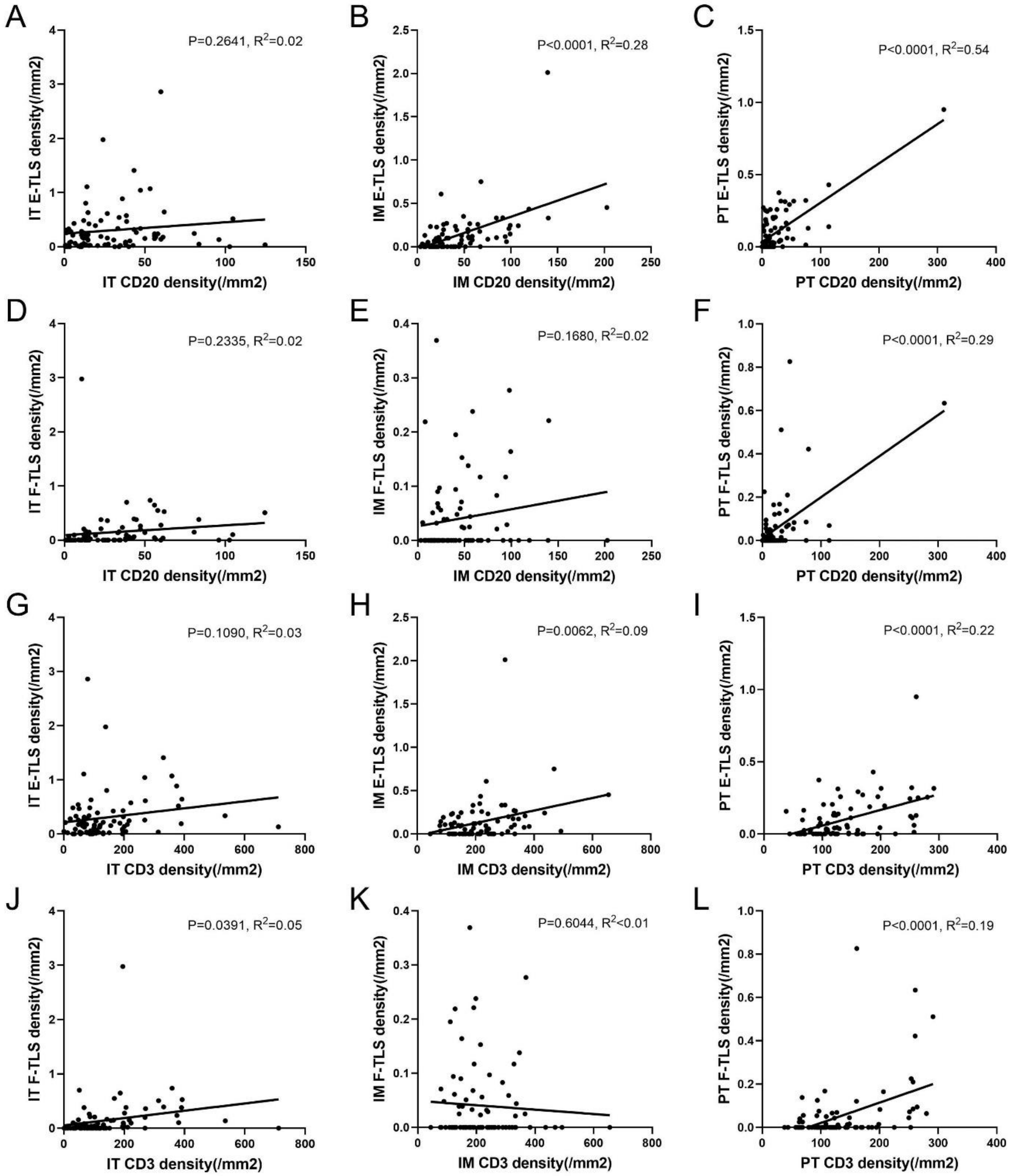

Figure S2. Association between E-TLS, F-TLS and immune cells. (A-C) General linear regression analysis was performed to determine the correlation between CD20+ B cells in IT, IM and PT regions and E-TLS density in the corresponding regions. (D-F) General linear regression analysis was performed to determine the correlation between CD20+ B cells in IT, IM and PT regions and F-TLS density in the corresponding regions. (G-I) General linear regression analysis was performed to determine the correlation between CD3+ T cells in IT, IM and PT regions and E-TLS density in the corresponding regions. (J-L) General linear regression analysis was performed to determine the correlation between CD3+ T cells in IT, IM and PT regions and F-TLS density in the corresponding

regions. IT: intratumor region, IM: invasive margin region, PT: peritumor region, F-TLS: secondary TLS, E-TLS: primary TLS.

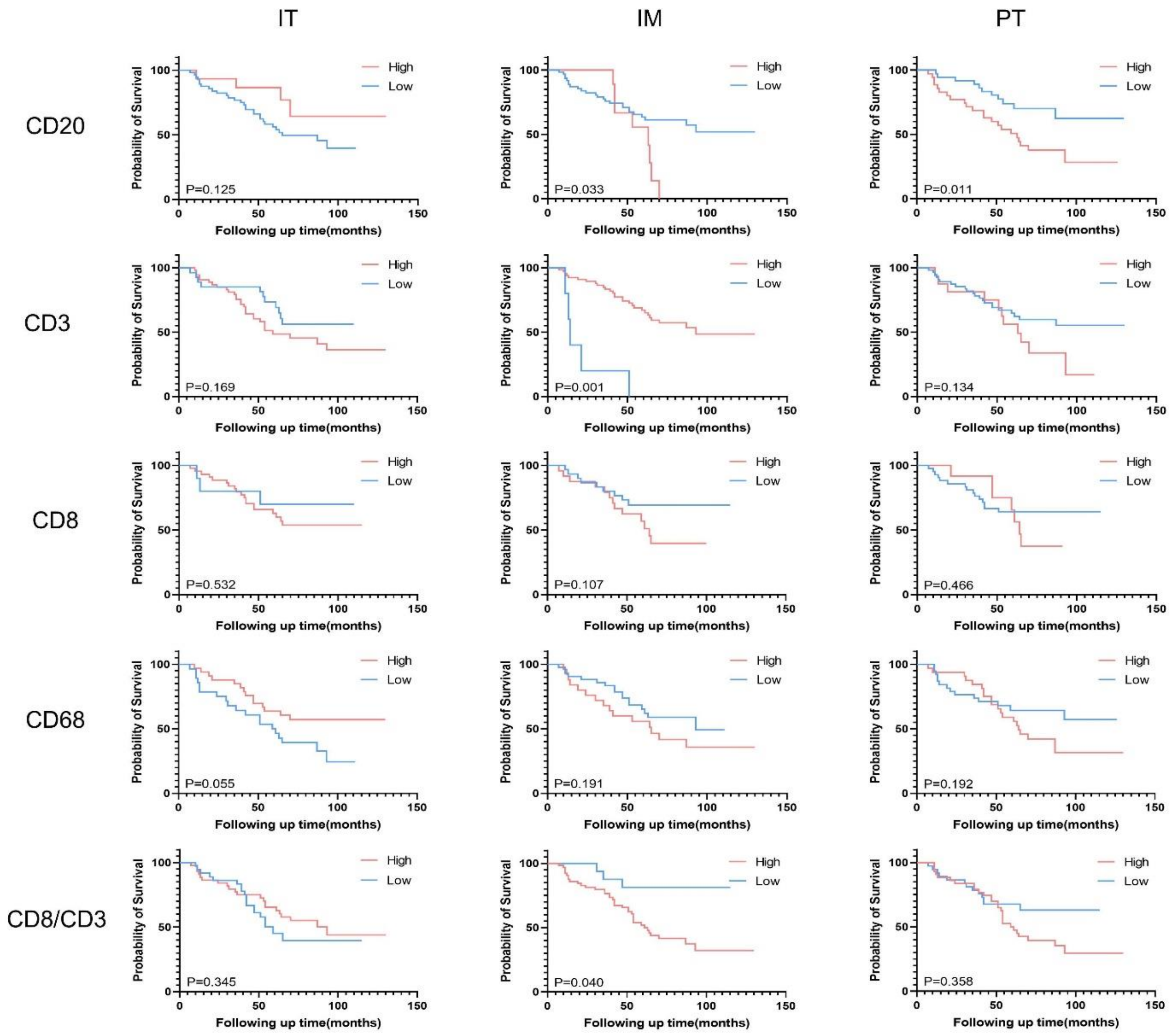

Figure S3. Correlation between the density of immune cells and overall survival of NSCLC patients. Immune cells were divided into groups of high expression and low expression according to ROC curve, and Kaplan Meier analysis was performed. IT: intratumor region, IM: invasive margin region, PT: peritumor region.
